# Platelet to lymphocyte ratio predicting 6-month primary patency of drug-coated balloon for femoropopliteal disease

**DOI:** 10.1186/s12872-019-01314-1

**Published:** 2020-01-09

**Authors:** Yanhua Zhen, Zhihui Chang, Zhaoyu Liu, Jiahe Zheng

**Affiliations:** Department of Radiology, Shengjing Hospital of China Medical University, Shenyang 110004, 36, Sanhao Street, Heping District, Shenyang City, China

**Keywords:** Platelet, Lymphocyte, Femoropopliteal disease, Drug-coated balloon, Primary patency

## Abstract

**Background:**

Inflammatory reaction is an essential factor in the occurrence, development and prognosis of femoropopliteal disease (FPD). The ratio of platelets to lymphocytes (PLR) is a new indicator reflecting platelet aggregation and burden of systemic inflammation. Our study is to explore the association between preoperative platelet-to-lymphocyte ratio (pre-PLR) and 6-month primary patency (PP) after drug-coated balloon (DCB) in FPD.

**Methods:**

There were 70 patients who underwent DCB for FPD contained in the study. According to 6-month PP, patients were divided into group A (PP ≥6 months, *n* = 54) and group B (PP < 6 months, *n* = 16). Logistic regression analysis was used to identify potential predictors for 6-month PP after DCB in FPD. A receiver operating characteristic (ROC) curve analysis was used to identify the cut-off value of pre-PLR to predict 6-month PP.

**Results:**

Logistic regression analysis showed that pre-PLR (OR: 1.008, 95% CI: 1.001–1.016, *P* = 0.031) and lesion length > 10 cm (OR: 4.305, 95% CI: 1.061–17.465, *P* = 0.041) were independently predictive for 6-month PP. The cutoff value of pre-PLR obtained from the ROC analysis was 127.35 to determine 6-month PP with the area of 0.839. Subgroup analysis was conducted based on the cutoff value of pre-PLR. The 6-month PP in the group of pre-PLR < 127.35 was higher than that of pre-PLR ≥ 127.35 group (*p* < 0.001).

**Conclusions:**

The present study indicated that an elevated pre-PLR was an effective additional indicator for predicting early PP in FPD after DCB.

## Background

A great deal of literature has shown that the inflammatory reaction is an essential factor in the occurrence, development and prognosis of femoropopliteal disease (FPD) [1–4]. Previous studies indicate that platelets are critical in the formation of atherosclerosis, not only as a mediator of thrombosis, but also as inflammatory cells, involved in immune-mediated plaque instability and the process of chronic atherosclerosis [5–7]. In addition, lymphocytes play a regulatory role in inflammatory response, and have a protective effect on atherosclerosis [8]. During the inflammatory response, the number of lymphocytes decrease due to accelerated apoptosis [9, 10].The ratio of platelets to lymphocytes (PLR) is a new indicator reflecting platelet aggregation and burden of systemic inflammation. It represents two opposite associations which are more stable relative to absolute platelet or lymphocyte counts [11]. Previous reports have shown that PLR is closely related to the severity of heart disease and the prognosis after interventional therapy [12, 13]. And for patients suffering from carotid artery stenosis, PLR was observed to be an independent variable to predict stroke [14]. However, the relationship between preoperative platelet-to-lymphocyte ratio (pre-PLR) and prognosis of patients with FPD undergoing percutaneous transluminal angioplasty (PTA) with drug-coated balloon (DCB) is unknown, it is supposed that pre-PLR was predictive of 6-month PP rate after DCB for FPD.

## Methods

The Shengjing Hospital of China Medical University institutional review board approved this retrospective study with a waiver of informed consent. Seventy patients who underwent DCB for FPD in our institution were contained in our analysis. The main exclusion criteria included in-stent stenosis and any diseases that were referred to infectious diseases, malignant tumor and autoimmune diseases.

Baseline clinical characteristics (age, man, hypertension, smoke, diabetes, hyperlipemia, cardiovascular disease), lesion characteristics (severe calcium, critical limb ischemia (CLI), total occlusion, outflow, lesion length), bail-out stent, the counts of preoperative white blood cell (WBC), mean platelet volume (MPV), platelets, lymphocytes, and neutrophils were obtained from medical records. Severe calcium was defined as calcium lesion≥180^。^ (or both sides of the vessel at the same location) and ≥ half total lesion length [15]. PLR was the ratio of absolute count of platelets to lymphocytes generated from the blood samples, and neutrophil-lymphocyte ratio (NLR) was the ratio of absolute count of neutrophils to lymphocytes.

All patients received dual antiplatelet therapy with acetylsalicylic acid (ASA) (100 mg/d) and clopidogrel (75 mg/d) for 5 days or a preload of 300 mg of ASA and clopidogrel before DCB. Pre-dilatation with uncoated balloon was conducted conventionally before DCB. The DCB used in this study was coated with paclitaxel at 3 μg/mm^2^, and the method of use was as described previously [16].

Follow-up with a primary patency (PP) at 6 months was the primary outcome, which was considered as no clinically driven target lesion revascularization or restenosis. Stenosis more than 50% by duplex ultrasound with a peak systolic velocity ratio > 2.4 was identified as restenosis.

### Statistical analysis

Data were calculated by SPSS 25.0 (SPSS Inc., Chicago, Illinois). Quantitative data were expressed as mean with standard deviation or median with interquartile, and qualitative data were expressed with frequencies and percentages. For normally distributed data comparisons were tested by Chi - square test or the Student t test. Analyses were performed using non-parametric statistical methods when variables showed markedly non-normal distribution. Significant univariate factors with *p* < 0.1 were further tested in the multivariable model. And odds ratio (OR) was used with 95% confidence interval (CI) to present potential predictors for 6-month PP. A receiver operating characteristic (ROC) curve analysis was constructed to judge the prediction ability of pre-PLR in 6-month PP. A two tailed *P* < 0.05 indicated statistical significance.

## Results

Patient characteristics were showed in Table 1. According to 6-month PP, we divided the patients into group A (PP ≥6 months, *n* = 54) and group B (PP <6 months, *n* = 16). Significant differences were observed between the two groups concerning lesion length > 10 cm, pre-platelets, pre-lymphocytes, MPV, pre-PLR (*p* < 0.05), no differences in other clinical and lesion characteristics were reported in the two groups (*p* > 0.05).

**Table 1:** Comparison of demographic and clinical characteristics of the 70 patients grouped by 6-month primary patency

	Group A^a^ (*n* = 54)	Group B^b^ (*n* = 16)	p
Man, n (%)	39(72.2)	15(93.8)	0.074
Age, mean (SD)	65.52(10.55)	68.38(8.46)	0.325
Hypertension, n (%)	37(68.5)	12(75.0)	0.622
Smoke, n (%)	42(77.8)	10(62.5)	0.223
Diabetes, n (%)	29(53.7)	12(75.0)	0.132
Hyperlipemia, n (%)	13(24.1)	5(31.3)	0.567
Cardiovascular disease, n (%)	21(38.9)	10(62.5)	0.097
Severe calcium, n (%)	5(9.3)	1(6.3)	1.000
CLI, n (%)	20(37.0)	10(62.5)	0.073
Total occlusion, n (%)	38(70.4)	11(68.8)	0.902
Outflow, n (%)			
1	10(18.5)	7(43.8)	
2	14(25.9)	3(18.8)	0.087
3	30(55.6)	6(37.5)	
Bail-out stent, n (%)	6(11.1)	2(12.5)	0.879
Lesion length > 10 cm, n (%)	20(37.0)	12(75.0)	0.008
WBC, median (IQR)	6.86(5.86–9.18)	6.62(5.01–9.34)	0.727
Platelets, mean (SD)	203.39(65.56)	249.13(75.45)	0.021
MPV, median (IQR)	9.00(8.37–9.80)	8.45(8.00–8.90)	0.070
Neutrophils, median (IQR)	4.35(3.6–6.0)	3.65(2.85–6.82)	0.511
Lymphocytes, median (IQR)	1.95(1.57–2.60)	1.50(1.20–1.78)	0.001
Pre-NLR, median (IQR)	2.14(1.70–2.78)	3.29(1.85–4.32)	0.055
Pre-PLR, median (IQR)	94.11(67.99–125.48)	187.29(131.67–212.86)	<0.001

Abbreviations: *SD* Standard deviation, *IQR* Interquartile range, *CLI* Critical limb ischemia, *WBC* White blood cell, *MPV* Mean platelet volume, *pre-NLR* Preoperative neutrophil-to-lymphocyte ratio, *pre-PLR* Preoperative platelet-to-lymphocyte ratio

^a^Primary patency≥6 months; ^b^Primary patency<6 months

On univariate analysis, pre-PLR, lesion length > 10 cm, CLI, MPV and outflow were significant prognostic factors, and multivariate logistic regression analysis showed that pre-PLR (OR: 1.008, 95% CI: 1.001–1.016, *P* = 0.031) and lesion length > 10 cm (OR: 4.305, 95% CI: 1.061–17.465, *P* = 0.041) were independently predictive for 6-month PP (Table 2).

**Table 2:** Logistic regression analysis for predicting 6-month primary patency

	Univariate analysis			Multivariate analysis		
	OR	CI	p	OR	CI	P
Man	5.769	0.669,47.587	0.104			
Age	1.029	0.972,1.089	0.321			
Hypertension	1.378	0.387,4.903	0.620			
Smoke	0.476	0.144,1.578	0.225			
Diabetes	2.586	0.740,9.042	0.137			
Hyperlipemia	1.434	0.420,4.892	0.565			
Cardiovascular disease	2.619	0.829,8.276	0.101			
CLI	2.833	0.894,8.975	0.077	1.992	0.446,8.286	0.381
Severe calcium	0.653	0.071,6.037	0.708			
Total occlusion	0.926	0.277,3.099	0.901			
Outflow (1)	3.500	0.95,12.898	0.060	2.360	0.494,11.282	0.282
Outflow (2)	1.071	0.233,4.919	0.929	0.631	0.096,4.159	0.632
Lesion length > 10 cm	5.100	1.448,17.965	0.011	4.305	1.061,17.465	0.041
Bailout-stent	1.143	0.207,6.303	0.878			
WBC	1.042	0.831,1.307	0.719			
Pre-NLR	1.210	0.960,1.524	0.106			
MPV	0.545	0.283,1.050	0.069	0.850	0.448,1.612	0.618
Pre-PLR	1.011	1.003,1.020	0.008	1.008	1.001,1.016	0.031

Abbreviations: *OR* Odds ratio, *CI* Confidence interval, *CLI* Critical limb ischemia, *WBC* White blood cell, *MPV* Mean platelet volume, *pre-NLR* Preoperative neutrophil-to-lymphocyte ratio, *Pre-PLR* Preoperative platelet-to-lymphocyte ratio

**Fig. 1:**
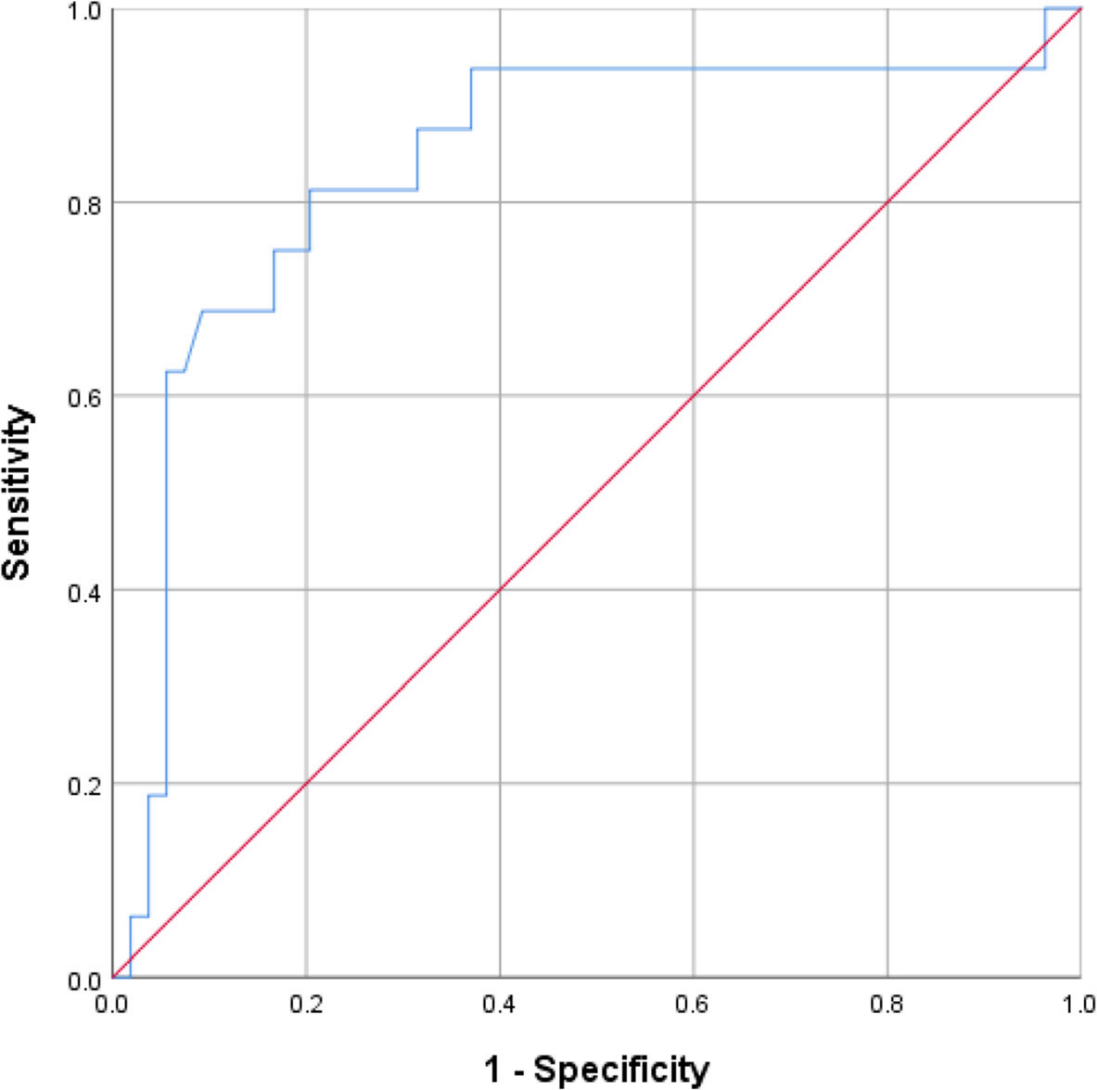
Receiver operating characteristic (ROC) curve for 6-month primary patency according to preoperative platelet-to-lymphocyte ratio. The area under the ROC curve was 0.839, and the sensitivity and specificity were 81.3 and 79.6%, respectively

The cutoff value of pre-PLR calculated from the ROC curve analysis was 127.35 for determination of 6-month PP. The area under the ROC curve was 0.839 (95% CI: 0.712–0.965, *P* < 0.001) with the sensitivity of 81.3% and specificity of 79.6%, respectively (Fig. 1).

Subgroup analysis was conducted based on the cutoff value of pre-PLR (127.35), the 6-month PP in the group of pre-PLR < 127.35 was higher than that of pre-PLR ≥ 127.35 group (*p* < 0.001).

## Discussion

In this study, we investigated the association between pre-PLR and the prognosis of FPD. The results indicated that pre-PLR and lesion length > 10 cm were independently predictive for 6-month PP after DCB for FPD. The cutoff value of pre-PLR was 127.35 with the sensitivity of 81.3% and specificity of 79.6%, respectively. Subgroup analysis showed that the 6-month PP in the group of pre-PLR < 127.35 was higher than that of pre-PLR ≥ 127.35 group.

Increased platelets and platelet activation are key factors in the development of thrombosis and atherosclerosis [17, 18]. When atherosclerotic plaque ruptures or blood vessels are damaged after PTA, platelets in this area adhere and aggregate, which promote the development of mural thrombus or occlusive thrombus, thus leading to the restenosis of lower limb artery disease [19, 20]. Closely related to these conclusions, in the current study, the count of platelets was higher in group B compared with group A. In addition, platelets can interact with other inflammatory cells, resulting in a cascade of inflammation and atherosclerosis at the vessel wall [6].

Low lymphocytes are common manifestations of acute inflammatory reaction [21]. It is known that lymphocytes participate in the whole process of the occurrence, development and prognosis of atherosclerosis [22]. The low lymphocyte count is related to reduced hemodynamics and aerobic capacity. Lymphocytes apoptosis was observed in atherosclerotic lesions including plaque growth, lipid core development, plaque rupture and thrombosis. Low lymphocyte count has been confirmed to be related to poor prognosis in patients with heart disease [23].In addition, lymphocytes play an important part in tissue healing, Iso et al. reported that lymphocyte count seems to be related to limb salvage rate of CLI patients [24].In the present study, lymphocytes in group B were lower than that of group A, which indicated that lymphocytes may be a protective factor in restenosis.

PLR represents two opposite associations which are superior to absolute platelet or lymphocyte counts. It is easy to perform in the blood test, and an increased PLR may yield further evidence on inflammatory response and the prothrombotic state [25]. It has been reported that the raised PLR was related to poor prognosis when patients suffered from coronary artery disease and aortic stenosis [12, 26–28]. In the present study, the pre-PLR was predictive of PP at 6 months, and the 6-month PP in the group of pre-PLR < 127.35 was higher than that of pre-PLR ≥ 127.35 group, which should also be noticed during the postoperative treatment.

NLR was already tested as a marker of the response of the immune system and predicted the presence of atherosclerotic plaques in elderly patients [29]. Li et al. [30] concluded that higher pre-NLR and pre-PLR levels were independent risk factors for the development of in-stent restenosis in patients who underwent drug-eluting stent implantation for coronary chronic total occlusion lesions. However, in the study, pre-NLR was not an independent predictor of PP at 6 months, which is in accordance with our previous report [16]. Larger samples are still needed to confirm our conclusion in the future.

It is known that DCB with antiproliferative drugs has the effect of inhibiting intimal hyperplasia and reducing the occurrence of restenosis [31]. However, in complex FPD including severe calcium, long segment lesions, the applications of DCB angioplasty are often excluded for lesions with flow-limiting dissection or high residual narrowing after pretreatment with uncoated balloon, and bail-out stenting has to be used in some cases [32]. In the present study, lesion length was divided into groups based on the median length, and lesion length > 10 cm was a risk factor in 6-month PP, while it seems that severe calcium has no influence in 6-month PP. It has been widely recognized that severe calcium can increase the technical difficulty and reduce the durability of endovascular therapy in the FPD [4, 15, 33], however there was not a standard classification of calcium degree till now. So, it is important to define an appropriate calcification grading system which can have a good relationship with the major procedure outcome.

Several limitations of this study deserve attention. First, this is a small size and single-center retrospective analysis which may lead to the selection bias and a type II error. Secondly, we did not dynamically monitor the changes in PLR after treatment. Finally, other inflammatory mediators in the blood sample were not involved in this study.

## Conclusion

The present study showed that pre-PLR was an independent risk factor in predicting 6-month PP. Pre-PLR, a simple and easy-to-obtain blood cell parameter, tends to be higher in the restenosis group after FPD receiving DCB angioplasty. Preoperative elevated PLR maybe helpful to identify those at risk of early restenosis and lay the groundwork for novel approaches for preventing restenosis.

## Data Availability

The raw data of this study will not be shared publically because they will be applied for further researches of this series but the datasets used and/or analyzed during the current study are available from the corresponding author on reasonable request.

## Abbreviations

pre-PLR: Preoperative platelet-to-lymphocyte ratio
PP: Primary patency
DCB: Drug-coated balloon
FPD: Femoropopliteal disease
PTA: Percutaneous transluminal angioplasty
pre-NLR: Preoperative neutrophil-lymphocyte ratio
WBC: White blood cell
MPV: Mean platelet volume
ASA: Acetylsalicylic acid
OR: Odds ratio
CI: Confidence interval
ROC: Receiver operating characteristic
CLI: Critical limb ischemia
